# The Complete Works of Hippocrates, &c.

**Published:** 1847-01

**Authors:** 


ART. XX.—*Œuvres Complètes d'Hippocrate, Traduction Nouvelle, avec le Texte Grec en regard, collationné sur les Manuscrits et toutes les Editions; accompagnée d'une Introduction, de Commentaires Medicaux, de Variantes, et de Notes Philologiques; suivie d'une Table Générale des Matières.* Par E. LITTRÉ, de l'Institut, de l'Académie des Inscriptions et Belles Lettres, et de la Société d'Histoire Naturelle de Halle.—*Paris,* 8vo, Vol. IV. 1844, pp. xx, 670; Vol. V. 1846, pp. 733.

*The Complete Works of Hippocrates, a new Translation, with the Greek Text on the opposite Pages, collated with the Manuscripts and all the Editions; with an Introduction, Medical Commentaries, various Readings, and Philological Notes; followed by a general Index of the matter therein contained.* By E. LITTRÉ, &c.

THIS work proceeds somewhat slowly, at least much more slowly than the learned Editor anticipated when seven years ago he published his first volume, and promised that the whole should be finished at the rate of three volumes a year. It goes on however steadily and satisfactorily, which is of more consequence; and we know by the experience of two or three memorable instances in this country that it is not easy to attain the punctuality of the Editors of the *Penny Cyclopædia* (and may we not add) the *Cyclopædia of Practical Medicine,* in works of equal magnitude.

The fourth volume which was published in 1844 finishes the list of the genuine works of Hippocrates, and contains the treatises "De Articulis," "Mochlicus," "Aphorismi," "Jusjurandum," and "Lex." In his "Avertissement" and his "Addenda et Corrigenda" the Editor takes the opportunity of noticing some points in the former volumes which required further consideration, and in some cases, with his usual candour, corrects and modifies his former statements. To each work he prefixes an "Argument," or introduction, in which he discusses (and sometimes at considerable length,) the subject matter of the treatise, and supplies such preliminary information as may be necessary for understanding it more thoroughly. Some of these introductions are extremely interesting, and contain a valuable store of antiquarian information, illustrated and explained by frequent reference to the present state of Medical Science. The volume closes with some "Remarques Rétrospectives," on the character of the genuine works of Hippocrates and the general ideas by which they were inspired.

The fifth volume contains the Second, Fourth, Fifth, Sixth, and Seventh books, "De Morbis Popularibus," "De Humoribus," the first book of the "Praedictiones," and the "Coacal Praenotiones;" all of which treatises are edited, translated, and explained in the same style as the preceding works. One of the most interesting discussions introduced in this volume is that on the meaning of the word ἄνθραξ, which introduces a digression on the history of the Smallpox. (p. 484.) The learned editor not only quotes the chronicles of Marius and Gregory of Tours to prove that this disease had appeared in Europe earlier than is commonly supposed, but also remarks of the plague of Athens, and of that in the reign of M.

Aurelius Antoninus, that, "if they were not identical with the Smallpox, they had at least considerable resemblances with that disease." Into this "vexata quæstio" we shall not enter at present, but shall content ourselves with once more recommending M. Littré's work to all our public libraries, and to those individuals who take an interest in ancient medical literature, as being the most important work of the kind in completeness, if not in extent, that has been undertaken for more than a century.

